# Bigels as Fat Replacers in Fermented Sausages: Physicochemical, Microbiological, Sensory, and Nutritional Characteristics

**DOI:** 10.3390/gels9040340

**Published:** 2023-04-16

**Authors:** Christina Siachou, Konstantina Zampouni, Eugenios Katsanidis

**Affiliations:** Department of Food Science and Technology, School of Agriculture, Faculty of Agriculture, Forestry and Natural Environment, Aristotle University of Thessaloniki, 54124 Thessaloniki, Greece

**Keywords:** olive oil bigels, fermented sausages, animal fat substitution, κ-carrageenan, monoglycerides, gelatin

## Abstract

Olive oil bigels structured with monoglycerides, gelatin, and κ-carrageenan were designed for the partial substitution of pork backfat in fermented sausages. Two different bigels were used: bigel B60 consisted of 60% aqueous and 40% lipid phase; and bigel B80 was formulated with 80% aqueous and 20% lipid phase. Three different pork sausage treatments were manufactured: control with 18% pork backfat; treatment SB60 with 9% pork backfat and 9% bigel B60; and treatment SB80 with 9% pork backfat and 9% bigel B80. Microbiological and physicochemical analyses were carried out for all three treatments on 0, 1, 3, 6, and 16 days after sausage preparation. Bigel substitution did not affect water activity or the populations of lactic acid bacteria, total viable counts, *Micrococcaceae*, and *Staphylococcacea* during the fermentation and ripening period. Treatments SB60 and SB80 presented higher weight loss during fermentation and higher TBARS values only on day 16 of storage. Consumer sensory evaluation did not identify significant differences among the sausage treatments in color, texture, juiciness, flavor, taste, and overall acceptability. The results show that bigels can be utilized for the formulation of healthier meat products with acceptable microbiological, physicochemical, and organoleptic characteristics.

## 1. Introduction

Meat products are considered as widely acceptable and liked foods for consumers, while there is a remarkable preference for fermented meat products. The latter have distinctive organoleptic properties which are attributed to the techniques applied for their preparation. Fermented products are subjected mostly to lactic acid bacteria fermentation and dehydration in order to ensure their microbiological safety and stability [1].

Fermented sausages are manufactured with minced meat and added animal fat which exists in the form of visible fat particles within the mass of the product. Animal fat plays a very essential role, affecting the fermentation process and the dehydration rate of sausages [2]. Furthermore, it is responsible for some specific organoleptic properties of meat products such as palatability, texture, and flavor [3].

Despite that, animal fat is rich in saturated fatty acids and cholesterol. Excessive consumption of such lipids is related to cardiovascular disease, obesity, and high levels of LDL (low density lipoprotein) cholesterol; hence, current nutritional guidelines of most health organizations recommend the limitation of such lipids [4,5].

In an attempt to address this matter, interest has shifted towards the manufacture of meat products with reduced fat content and improved fatty acid profile. In this regard, fat substitutes with edible vegetable oils such as oleogels and emulsion gels have been developed [6,7,8].

Over the past few years, research on meat products with fat substitutes and their properties has intensified [9,10]. In the case of fermented meat products, there is a limited number of studies utilizing structured oil systems as fat substitutes. Monoglycerides—olive oil oleogels were applied as pork backfat substitutes in fermented sausages [11]. In other studies, olive and chia oil oleogels and emulsion gels [12] and inulin-based emulsion-filled gels [13] were applied in dry fermented sausages for the partial replacement of pork backfat. Based on these studies, it is deduced that the concept of healthier meat products with fat substitutes, produced with vegetable oils, is very promising and should be further investigated.

Recent studies have focused on the utilization of structured systems such as bigels or hybrid gels as fat substitutes. Bigels are semi-solid structured systems with a lipophilic and a hydrophilic phase [14]. One phase is dispersed and the other is continuous and the bigel structure is obtained by mixing both phases under high shear rate in suitable temperatures [15]. The lipid phase is converted into an oleogel, structured by various oleogelators. The aqueous phase forms a hydrogel with the appropriate hydrophilic gelling agents which are capable of absorbing large quantities of water [16,17]. Oleogels and hydrogels are generally thermoreversible systems. Bigels, however, due to their biphasic nature, do not usually exhibit a similar behavior, something that may constitute a limitation in cooked food products. Several bigel matrices have been researched with different vegetable oils and gelators, such as sesame oil bigel with sorbitan monostearate and guar gum [18], sunflower oil and protein-based bigel [19], bigel with rice bran oil, stearic acid, and tamarind gum [20], and olive oil bigels with monoglycerides and gelatin/κ-carrageenan [21].

Bigels can act as delivery systems for both hydrophobic and hydrophilic molecules and bioactive compounds modulating their release profile, such as β-carotene, lycopene, or rosemary extract [22,23,24,25]. Until now, bigels have been developed and analyzed mostly for cosmetic and pharmaceutical purposes and there are only a few studies related to the development of edible bigels [26,27,28]. Therefore, the potential application of bigels in food systems and especially in meat products as fat substitutes is a very interesting and promising research subject.

The objective of the present study was the formulation of fermented sausages with partial substitution of pork backfat by bigels structured with olive oil, monoglycerides, gelatin and κ-carrageenan. Analysis of the physicochemical, microbiological, organoleptic, and nutritional properties of the sausages was performed in order to evaluate the functionality of the olive oil bigel during sausage formulation, fermentation, and dehydration.

## 2. Results and Discussion

### 2.1. Physicochemical Analysis

#### 2.1.1. Weight Loss

**Figure 1:**
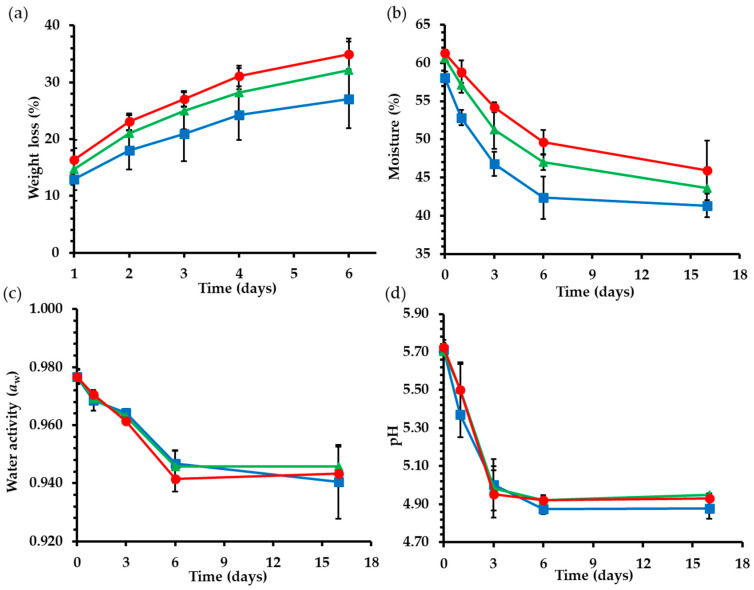
Weight loss % (**a**), moisture % (**b**), water activity (a_w_) (**c**), and pH (**d**) during fermentation and ripening of fermented sausages. (■) Control: 18% pork backfat; (▲) SB60: 9% pork backfat and 9% B60 bigel; (●) SB80: 9% pork backfat and 9% B80 bigel.

During fermentation and ripening of the sausages, all treatments lost weight due to dehydration (Figure 1a). Dehydration, combined with salt addition, contributes to a significant reduction in water activity, conferring microbial stability and, as a result, a satisfactory shelf life of the product. In addition, this process improves the mechanical and organoleptic properties of the sausages. Loss of moisture is affected by the prevailing conditions in the fermentation chamber, such as temperature, relative humidity, and air velocity [29]. Additionally, the composition and structure of the fat replacer could also have an impact on the drying process of the sausages. According to the results, fat substitution by olive oil bigel had a significant effect (*p* < 0.05) on sausages’ weight loss. In particular, the control treatment had lower weight loss than treatments SB60 and SB80. During fermentation, weight loss of control treatment increased from 12.91 to 27.07%. Treatment SB80 presented higher weight loss values (16.37–34.90%) in comparison with SB60 (14.71–32.16%). Despite this fact, treatments SB60 and SB80 still had higher moisture content compared to control samples at the end of the dehydration and fermentation procedure.

Higher weight loss can be related to the higher water content of samples that were formulated with bigels. B80 bigels were prepared with a higher water content than B60 bigels; specifically, SB60 samples contained 4.65% and SB80 samples 5.52% more water than the control treatment. This amount of water is immobilized within the structure of the bigels. Similar findings were reported by researchers, who studied the application of konjac gel as a pork backfat substitute in fermented sausages [30]. Moreover, higher weight loss values in sausages with linseed oil-gelled emulsion—due to the extra water present in the emulsion— have been reported [31]. In addition, in studies with reduced-fat fermented sausages, it is reported that samples with a higher fat content had lower weight loss, since animal fat contributes to a gradual and controlled release of moisture [32,33].

#### 2.1.2. Moisture Content

During the fermentation procedure, sausage samples are subjected to dehydration; thus, there was a gradual decrease in their moisture content. The different sausage treatments had slightly different initial moisture contents due to the different moisture contents of pork backfat and the bigels. The initial moisture content on day 0 was 58% for the control treatment, 60.7% for SB60, and 61.2% for SB80. After the dehydration on day 6, when the fermentation and ripening process was completed, the water content decreased to 42.3% for control, 47% for SB60, and 49.6% for SB80 samples (Figure 1b). The higher final moisture content was observed for SB60, as expected. Control treatments had significantly lower moisture content (*p* < 0.05) compared to treatments SB60 and SB80. These findings are consistent with previous studies that investigated the formulation of fermented meat products with pre-emulsified fat and emulsion gels [13,32]. Higher moisture content values of treatments SB60 and SB80 are attributed not only to the fact that they were manufactured with higher water content, but also because the bigel structure is incorporated in the meat matrix and remains intact during dehydration, resulting in the binding of the water into the gel network.

#### 2.1.3. Water Activity (α_w_)

Fermented meat products are characterized by low water activity as they are subjected to gradual dehydration in combination with the added salt. In this way, water is not available for the growth of undesirable microorganisms [34]. Partial fat replacement by bigels in the present study did not significantly affect the water activity of fermented sausages (*p* > 0.05). During the first 6 days, water activity values decreased from 0.977 to 0.941–0.946 because of dehydration (Figure 1c).

A previous study for gelatin hydrogels with carrageenan and potassium sulfate identified the presence of bound, immobilized, and free water inside the hydrogel network. Specifically, an increase in carrageenan percentage caused the formation of a gel with a more stable structure and denser network. As a result, there was a decrease in free water mobility [35]. In accordance with this assertion, the additional quantity of water in treatments SB60 and SB80 is strongly entrapped into the bigel network, and it is possibly characterized by low mobility and, consequently, by low water activity. In this way, samples SB60 and SB80 presented similar water activity values to the control samples. In contrast with these findings, other researchers who formulated fermented sausages with inulin and linseed oil emulsion gel found that modified samples had significantly higher water activity values than control samples [13].

#### 2.1.4. pH

Lactic acid bacteria cause a decrease in pH values as they metabolize sugars that are available in the meat mixture and produce lactic acid during the fermentation process [36]. A rapid decrease in pH values from 5.70–5.73 to 4.95–5.00 was observed during the first 3 days of fermentation. A further slight decrease in pH values was also observed until day 6 (Figure 1d). The pork backfat replacement by bigels in fermented sausages significantly affected pH values (*p* < 0.05). Despite this, the observed differences in pH values were not considered as practically significant since the fermentation process and lactic acid production evolved normally for all three treatments. Regarding the observed pH differences, it is hypothesized that the presence of gelatin in the food matrix may have offered a slight buffering effect, preventing the drop in pH to the same extent. Zampouni et al. (2022) reported that fermented sausages formulated with olive oil and monoglycerides oleogel did not present significantly different pH values compared to control sausages [11]. In addition, it is reported that sausages prepared with carrageenan and zein/carboxymethyl dextrin emulsion gels did not exhibit significantly different pH values in comparison with the control treatment. This finding is attributed to the fact that fat substitute and pork backfat had similar pH values [37]. Similar findings are reported by Alejandre et al. (2016), who evaluated the application of linseed oil emulsion gels in dry fermented sausages [31]. On the contrary, some previous studies reported lower pH values for fermented sausages with fat substitution by emulsion gels and oleogels compared with control samples. Franco et al. (2019) reported that sausages with beeswax linseed oil oleogels presented lower pH values than control samples. This difference was attributed to the acidity of the oleogelator mixture compared to animal fat [38]. Moreover, Glisic et al. (2018) claimed that fermented sausages with inulin oleogels presented decreased pH values in comparison with controls due to the degradation of inulin by lactic acid bacteria [13].

#### 2.1.5. Lipid Oxidation

**Figure 2:**
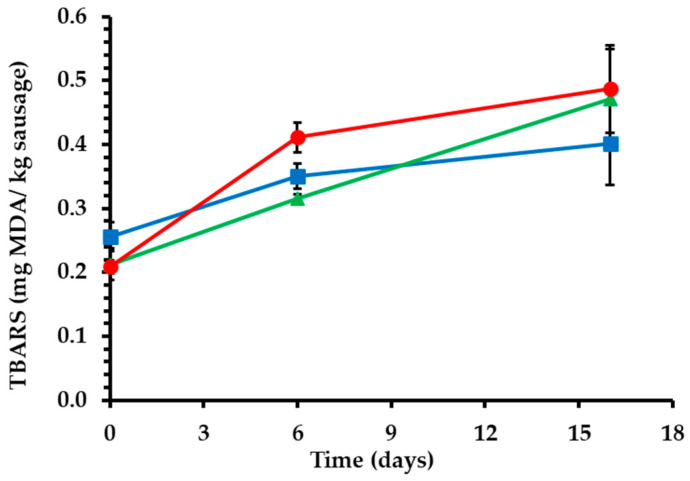
Oxidation values (TBARS) during fermentation and ripening of fermented sausages. (■) Control: 18% pork backfat; (▲) SB60: 9% pork backfat and 9% B60 bigel; (●) SB80: 9% pork backfat and 9% B80 bigel.

During the preservation of meat products, lipid oxidation can cause quality deterioration and reduce their shelf-life. In fact, lipid oxidation can reduce the nutritional value of meat products by causing the destruction of essential fatty acids and vitamins and can affect the organoleptic characteristics due to the appearance of a rancid odor and flavor. Moreover, lipid oxidation has been implicated in the production of toxic and harmful substances that have been related to chronic diseases [39,40]. During sausage fermentation and ripening, all three treatments presented an increase in oxidation levels (Figure 2). Treatments SB60 and SB80 presented significantly higher (*p* < 0.05) TBARS values than the control on the 16th day of storage. Bigels that partially substituted pork backfat in the present study were formulated with olive oil. Olive oil has a high content of unsaturated fatty acids, which are susceptible to lipid oxidation. Moreover, during bigel preparation, olive oil was heated at 90 °C for 30 min, and this thermal stress possibly accelerated the lipid oxidation process [41]. In addition, SB60 treatment presented higher TBARS values than the SB80 treatment, as it contained a higher ratio of olive oil oleogel in the bigel system. Similar results have been reported for dry fermented sausages with olive and chia oil oleogels and emulsion gels [12]. Moreover, bologna sausages formulated with soybean oil and inulin emulsion gels presented higher oxidation values than samples with animal fat, since soybean oil has a high content of unsaturated fatty acids [7]. It is important to mention that although the application of bigels increased oxidation values, the oxidation levels did not exceed the acceptable limits (<1) at any time during processing and storage.

### 2.2. Microbiological Analysis

**Figure 3:**
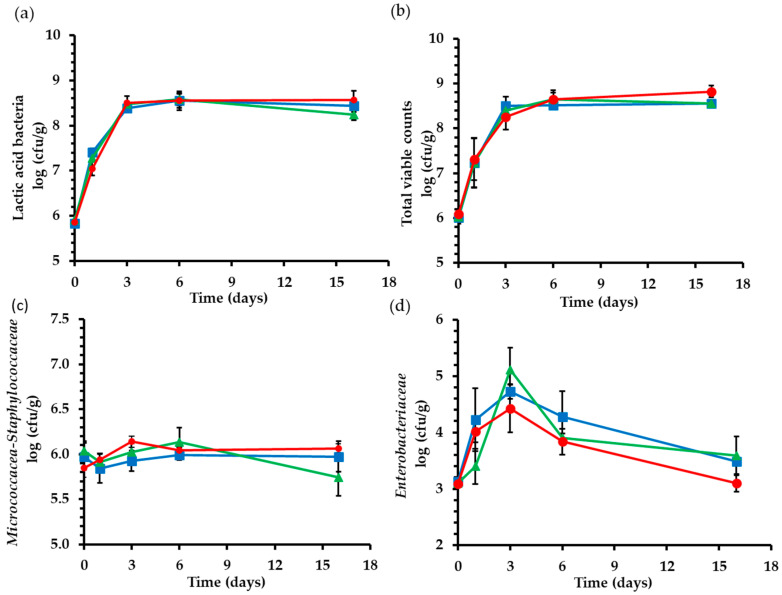
Lactic acid bacteria (**a**), Total viable counts (**b**), *Micrococcaceae-Staphylococcaceae* (**c**), and *Enterobacteriaceae* (**d**) counts during fermentation and ripening of fermented sausages. (■) Control: 18% pork backfat; (▲) SB60: 9% pork backfat and 9% B60 bigel; (●) SB80: 9% pork backfat and 9% B80 bigel.

Bigel substitution did not significantly affect the population of lactic acid bacteria, total viable counts, *Micrococcaceae*, and *Staphylococcaceae* in fermented sausages during the fermentation and ripening process (*p* > 0.05). During the first 3 days of the fermentation process, lactic acid bacteria populations presented a rapid increase from 5.5 to 8.5 log (cfu/g) for all three treatments (Figure 3a). A rapid increase of lactic acid bacteria and a significant decrease in pH values were detected simultaneously, indicating the beginning of the fermentation procedure [42]. Similar findings have been reported in a study regarding fat replacement in fermented sausages with olive oil used either as liquid or as pre-emulsified fat [32] and in a recent study that evaluated the application of olive oil oleogels in fermented sausages [11]. Total viable counts presented an increase from 6 log (cfu/g) to 8.6–8.8 log (cfu/g) on the 16th day (Figure 3b). Similar evolution for the population of the total viable counts was reported in fermented sausages by other researchers [43]. The populations of *Micrococcaceae*/*Staphylococcaceae* remained stable between 5.7 and 6.0 log (cfu/g) (Figure 3c). Zampouni et al. (2022) also reported a similar evolution of *Micrococcaceae*/*Staphylococcaceae* populations in sausages with olive oil oleogel as fat substitute [11].

Control treatments exhibited higher counts of *Enterobacteriaceae* (*p* < 0.05) in comparison with SB60 and SB80 treatments, but these differences are not greater than 0.5 log (cfu/g); thus, they are not evaluated as practically significant. The *Enterobacteriaceae* population increased until the third day, approaching a peak of 4.4–4.7 log (cfu/g) (Figure 3d). After that, a rapid decrease was observed due to the growth of the lactic acid bacteria and the production of lactic acid in combination with the decrease in *α*_w_ [44]. The observed differences in the *Enterobacteriaceae* populations may be attributed to slight differences in the initial microbial load of the lard and the population of microorganisms that act as competitive microflora. In addition, pH and *α*_w_ values combined with competitive microflora could have affected *Enterobacteriaceae* growth. In an analogous study, Glisic et al. (2018) substituted animal fat in fermented sausage with inulin emulsion gel and linseed oil and reported that fat substitution had no significant effect on the *Enterobacteriaceae* during the fermentation process [13].

### 2.3. Sensory Evaluation

**Figure 4:**
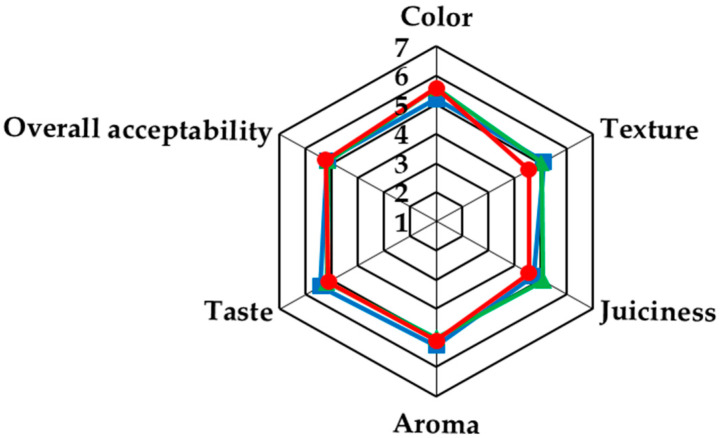
Sensory analysis scores for color, texture, juiciness, aroma, taste, and overall acceptability of the fermented sausages. (■) Control: 18% pork backfat; (▲) SB60: 9% pork backfat and 9% B60 bigel; (●) SB80: 9% pork backfat and 9% B80 bigel.

Sensory evaluation by consumers was carried out on cooked sausages. According to consumers’ evaluation, non-significant differences were detected among the treatments in color, texture, juiciness, flavor, and taste (*p* > 0.05). In addition, the overall acceptability of the substituted sausage treatments did not differ from that of the control (Figure 4). It is important to mention that although treatments SB60 and SB80 presented higher weight loss and moisture values than the control treatment, consumers did not detect any significant differences in the organoleptic characteristics of the three treatments. Thus, these results suggest that bigels can substitute the pork backfat up to 50% without negatively affecting the main organoleptic attributes of fermented sausages.

### 2.4. Nutritional Evaluation

The nutrient composition of the different sausage treatments is presented in Table 1. The addition of bigels as a fat substitute modified the sausages’ nutrient content, leading to changes in energy, protein, fat, saturated fatty acids, and cholesterol levels. It is worth noting that the decrease in energy and total fat per 100 g of the finished sausages with bigels can mainly be attributed to the different degrees of dehydration and the lower fat content of the bigels compared to pork backfat. According to the composition, the energy content of the control fermented sausages was approximately 376 kcal/100 g. As a result of the strategy to improve the nutritional value of the sausages, the energy content of the sausages with bigels decreased to 320 kcal/100 g for SB80, and 342 kcal/100 g for SB60. These changes correspond to an energy decrease of 9–15% in the reformulated finished products. Similar results for decreased energy were reported for dry fermented sausages either with linseed oil gelled emulsion [31], or olive oil in combination with chia oil emulsion gel as a fat replacer [12]. Regarding fat reduction, SB60 and SB80 showed 15.08% and 23.83% lower fat content, respectively, compared to the control. The higher reduction of total fat in SB80 results from the added bigel (B80) composition, mainly formulated by a hydrogel phase. Accordingly, there was a decrease of 20.53% (SB60) and 26.82% (SB80) in saturated fatty acids compared to the control. Moreover, the treatments with pork backfat substitution presented higher protein levels compared to the control (*p* > 0.05). Therefore, pork backfat replacement by bigels yielded a significant improvement in the nutritional profile of the fermented sausages.

## 3. Conclusions

Two bigels with different olive oil content were formulated as pork backfat substitutes in fermented sausages. The application of olive oil bigels did not affect the sausage fermentation process since no differences were detected in water activity and microbial populations of the different sausage treatments. A higher water loss can be attributed to the initial higher moisture content of the bigels. Although the bigel treatments had higher oxidation levels on the last day of storage, lipid oxidation was relatively low in all treatments, and it did not exceed the acceptable limits. In addition, partial animal fat substitution by olive oil bigels in fermented sausages did not affect their organoleptic characteristics, since all the sausage treatments were equally acceptable by the consumers. The application of bigels for the formulation of meat products with improved nutritional characteristics is a promising and interesting technique. Further research is required to assess the applicability of bigels in different kinds of meat products.

## 4. Materials and Methods

### 4.1. Bigel Preparation

For the purpose of the present study, two bigels with different hydrogel-to-oleogel ratios were prepared, namely, 60:40 (B60) and 80:20 (B80). The hydrogel phase of the bigels were formulated with 1% *w*/*w* κ-carrageenan and 10% *w*/*w* gelatin. The oleogel phase of the bigels was structured with 15% *w*/*w* monoglycerides in olive oil. The bigel formulations were selected based on previous research [21]. The main criterion was the consistency of the bigels, so that they can be successfully incorporated in the meat matrix of the sausage. To formulate the bigels, the lipid and aqueous phases were prepared separately. The oleogel was prepared by dissolving 15% *w*/*w* monoglycerides (HARI 95 Riketa SDN BHD, Johor Bahru, Malaysia) in preheated olive oil (Minerva SA, Metamorfosi, Greece). For hydrogel preparation, 1% *w*/*w* κ-carrageenan (Sigma-Aldrich Co., St. Louis, MO, USA) was dissolved into deionized water with 0.1 M KCl (Chem-Lab NV, Zedelgem, Belgium). Then, 10% *w*/*w* gelatin (ARA-CRISTAL-SUPER, RAPS Gmbh and Co. KG, Kulmbach, Germany) was added gradually, and the mixture was heated under stirring at 80 °C for 10 min. To formulate the bigels, the oleogel and hydrogel phases were mixed in the desired proportions under stirring at 80 °C for 15 min. Subsequently, the bigels were cooled in an ice-water bath for 45 min to set and obtain the desirable gel-like texture [21]. Then, the bigels were stored at 5 °C for 24 h until used for sausage formulation.

### 4.2. Sausage Preparation

For sausage formulation, fresh pork loin and pork backfat were purchased from the local market (Thessaloniki, Greece). Three different sausage treatments were manufactured. The control treatment (C) was formulated with 70.5% pork meat and 18% pork backfat. Moreover, two treatments with 50% pork backfat substitution were produced, each containing 70.5% meat, 9% pork backfat, and 9% bigel. Sausage treatment SB60 was formulated with B60 bigel, and treatment SB80 was formulated with B80 bigel. In all three treatments, common ingredients were added (fresh leek, salt, spices, and starter culture), as shown in Table 2. The starter culture consisted of *Pediococcus pentosaceus* and *Staphylococcus carnosus* (Bacto-Flavor^®^, BFL-T03, Chr. Hansen, GmbH, Pohlheim, Germany), and it was kept at −18 °C until sausage preparation. All sausages were filled into natural casings from pork small intestines with 40 mm diameter. After sausage production, each sample was weighed. The initial weight of each sausage was 250–300 g. Then, the sausages were placed in a controlled temperature and humidity chamber for fermentation and ripening for 6 days. The chamber’s temperature was 20 °C and the relative humidity was 85% for the first 3 days and 75% for the next 3 days. After the 6-day fermentation period, the sausage samples were vacuum-packaged individually and stored at 4 °C until further analysis. Sample analysis was conducted on days 0, 1, 3, 6 and 16.

### 4.3. Physicochemical Analysis

#### 4.3.1. Weight Loss

Each sausage sample was weighed right after their preparation procedure (day 0). A representative number of samples for each treatment were reweighed on days 1, 2, 3, 4, and 6. Weight loss was evaluated as the (%) difference between the initial measurement of weight and the measurement taken each day of sampling.

#### 4.3.2. Moisture

Moisture was measured following a standard method AOAC [45]. Weighed sausage samples of each treatment were subjected to air drying at 105 °C for 18–24 h until constant weight. Moisture was measured on days 0, 1, 3, 6, and 16 and determined in duplicate for each sausage treatment.

#### 4.3.3. Water Activity (a_w_)

The determination of water activity was carried out on days 0, 1, 3, 6, and 16 with a Water Activity Meter (Model series 3, Aqualab, Decagon Devices Incorporation, Pullman, DC, USA). Water activity was measured at room temperature. Duplicate water activity measurements were run for each treatment.

#### 4.3.4. pH

For the pH determination, 20 g of the sample was homogenized with 80 mL of distilled water using an Ultra Turrax T18 basic electric homogenizer (IKA, Staufen, Germany) equipped with an S18N-19G knife. The pH measurement was performed with HI 221 microprocessor pH meter (Hanna Instruments, Woonsocket, RI, USA) equipped with a glass electrode. The final pH value was obtained from the average of three separate measurements.

#### 4.3.5. Lipid Oxidation

Lipid oxidation was measured on days 1, 6, and 16 of sausage manufacture. It was determined according to a method based on the detection of secondary products of oxidation via thiobarbituric acid (TBA) by distillation with a stream of water vapor [46]. Specifically, 10 g of the sample and 25 mL of distilled water were weighed and transferred to a Duran flask. Then, the mixture was homogenized for 1–2 min with an Ultra Turrax T18 basic electric homogenizer (IKA, Staufen, Germany) equipped with an S18N-19G knife at 14,000 rpm. The mixture was then transferred into a small distillation flask with 5 mL 2 N HCl (Merk KGaA, Darmstadt, Germany) and 3–4 drops of silicon antifoam solution (Sigma-Aldrich, St. Louis, MO, USA). The sample was distilled with a stream of water vapor in a distillation unit (UDK 127, VELP Scientifica, Monza, Italy) until 50 mL of distillate were collected in a volumetric flask. Subsequently, 5 mL of the distillate with 5 mL of 0.02 M TBA solution (Alfa Aesar, Karlsruhe, Germany) was transferred to a capped test tube. All tubes were placed in a boiling water bath for 35 min and then cooled fast. Finally, the absorbance of the samples (A532) was measured at 532 nm on a Shimadzu UV-1700 spectrophotometer (Shimadzu Europe GmbH, Duisburg, Germany).

### 4.4. Microbiological Analysis

Microbiological analyses were carried out on days 0, 1, 3, 6 and 16. For each sausage treatment, 25 g of the sample was collected under aseptic conditions, transferred to a sterile stomacher bag with 225 mL sterile Ringer (Ringer Solution ¼ Strength, Lab M Limited, Lancashire, UK), and homogenized in a stomacher mixer for 1–2 min (BagMixer 400, Interscience, St. Nom, France). Then, the required serial dilutions were prepared and plated in duplicate into petri dishes. Total viable counts were enumerated with Plate Count Agar (PCA, Lab M Limited, Lancashire, UK) at 30 °C for 48 h, lactic acid bacteria with de Man Rogosa Sharpe Agar (MRS, Lab M Limited, Lancashire, UK) at 30 °C for 72 h, *Micrococcaceae* and *Staphylococcaceae* with Mannitol Salt Agar (MSA, Lab M Limited, Lancashire, UK) at 30 °C for 72 h, and *Enterobacteriaceae* with Violet Red Bile Agar (VRBGA, Lab M Limited, Lancashire, UK) at 37 °C for 24 h. Microorganisms populations were expressed as the decimal logarithm of the number of colony forming units per g of sample (log10 (cfu/g)).

### 4.5. Sensory Evaluation

A preference test was conducted to assess consumer liking and acceptance. This sensory evaluation session was carried out with the participation of 40 untrained panelists on the 10th day after sausage manufacture. Samples of the three sausage treatments were cut into cylindrical pieces (15 mm height and 40 mm diameter), grilled at 150 °C for 2 min at each side, and placed on white plates with random 3-digit codes. Evaluators were asked to assess the three treatments by evaluating color, texture, juiciness, flavor, and overall acceptability using a 7-point scale. In this scale, 1 corresponded to “very undesirable” and 7 to “very desirable”.

### 4.6. Nutritional Profile Calculations

The nutritional characteristics of the three sausage treatments were determined. The calculations were based on the initial composition of each treatment, considering the dehydration of the samples during the fermentation and ripening process. Nutrient data from the United State Department of Agriculture database [47] were used for these calculations.

### 4.7. Statistical Analysis

Fermented sausages were prepared in duplicate, in two separate experiments, with different raw materials each time. Analysis of the samples in each experiment were carried out in duplicate for each physicochemical and microbiological parameter. Data collected from the physicochemical, microbiological, and sensory evaluation analysis were analyzed by ANOVA using the General Linear Model. Mean values were compared using Tukey’s test for a 95% confidence interval using the Minitab 16.1.1 statistical package (Minitab, Inc., State College, PA, USA).

## Figures and Tables

**Table 1:** Nutritional composition and changes in the energy, fatty acids, cholesterol, and protein of uncooked fermented sausages per 100 g.

	Treatments		
Nutrient	Control	SB60	SB80
Water (g)	41.31 ± 1.40 ^a^	43.64 ± 2.21 ^a^	45.91 ± 3.10 ^a^
Energy (kcal/kJ)	376 ± 9 ^a^/1578 ± 38 ^a^	342 ± 13 ^a^/1438 ± 57 ^a^	320 ± 18 ^a^/1342 ± 77 ^a^
Total lipid (Fat) (g)	30.75 ± 0.74 ^a^	26.12 ± 1.03 ^b^	23.42 ± 1.34 ^b^
Fatty acids, total saturated (g)	11.03 ± 0.26 ^a^	8.77 ± 0.34 ^b^	8.07 ± 0.46 ^b^
Fatty acids, total monounsaturated (g)	14.37 ± 0.34 ^a^	13.35 ± 0.52 ^ab^	11.49 ± 0.66 ^b^
Fatty acids, total polyunsaturated (g)	3.54 ± 0.08 ^a^	2.65 ± 0.10 ^b^	2.51 ± 0.14 ^b^
Carbohydrate (g)	1.70 ± 0.14 ^a^	1.95 ± 0.07 ^a^	1.99 ± 0.12 ^a^
Fiber, total dietary (g)	0.24 ± 0.00 ^a^	0.25 ± 0.01 ^a^	0.25 ± 0.01 ^a^
Protein (g)	21.45 ± 0.52 ^a^	23.29 ± 0.92 ^a^	23.80 ± 1.57 ^a^
Sodium (mg)	919 ± 22 ^a^	983 ± 39 ^a^	988 ± 57 ^a^
Ash (g)	1.39 ± 0.03 ^a^	1.41 ± 0.06 ^a^	1.42 ± 0.08 ^a^
Cholesterol (mg)	85.13 ± 2.04 ^a^	83.21 ± 3.27 ^a^	83.62 ± 4.80 ^a^
Energy reduction (%)		8.89	14.91
Total fat reduction (%)		15.08	23.83
Saturated fatty acids reduction (%)		20.53	26.82
Cholesterol reduction (%)		2.25	1.77
Protein increase (%)		8.57	11.00

Values represent means ± standard deviation of the two experiment replications. Different letters in the same row (nutrient) indicate significant differences among treatments (*p* < 0.05). Control: 18% pork backfat; SB60: 9% pork backfat and 9% B60 bigel; SB80: 9% pork backfat and 9% B80 bigel.

**Table 2:** Composition (g/100 g of meat mixture) of the different fermented sausage formulations.

	Treatments		
Ingredients	Control	SB60	SB80
Pork meat	70.5	70.5	70.5
Pork backfat	18	9	9
Bigel	0	9	9
Leek	9	9	9
NaCl	1.5	1.5	1.5
Spices	0.99	0.99	0.99
Starter culture	0.01	0.01	0.01

## Data Availability

The data presented in this study are available on request from the corresponding author.
